# Meals, mealtimes and moments for learning: assessment of quality in early childhood education and care services

**DOI:** 10.1017/S1368980025000011

**Published:** 2025-01-07

**Authors:** Bonnie Searle, Sandy Houen, Sally Staton, Karen Thorpe

**Affiliations:** 1 Queensland Brain Institute, The University of Queensland, 79 Upland Road, St Lucia, QLD 4067, Australia; 2 Australian Centre of Excellence for Children and Families across the Life Course, The University of Queensland, 80 Meier’s Rd, Long Pocket, QLD 4068, Australia

**Keywords:** ECEC, Childcare, Mealtimes, Food provision, Interactions, Child development

## Abstract

**Objective:**

Early education and care (ECEC) is part of the everyday life of most children in developed economies, presenting exceptional opportunity to support nutrition and ongoing food preferences. Yet, the degree to which such opportunity is captured in policy-driven assessment and quality ratings of ECEC services is unknown.

**Design:**

Abductive thematic analysis was conducted, guided by key domains of knowledge in nutrition literature and examining identified themes within these domains.

**Setting:**

ECEC services (*n* 38) in Queensland, Australia.

**Participants:**

Data were a random sample of field notes pertaining to mealtimes and food provision (*n* 182) collected as evidence to inform quality ratings during assessment visits to ECEC services.

**Results:**

The field notes mapped to three theory-driven domains: *provisions, practices and education*. Reflecting policy specification, health, hygiene and safety were a key focus, but food quality and quantity were not. Assessors noted the promotion of child autonomy at mealtimes, yet little evidence pertaining to characteristics of educator-child interactions.

**Conclusions:**

Despite evidence that childhood nutrition is crucial for optimal development and learning, the quality and quantity of food are not directly assessed. Relationships and interactions at mealtimes provide an environment ideal for promoting learning and development, yet the policy guiding inspection and assessment of ECEC services directs focus to a more limited lens of safety, hygiene and promotion of ‘healthy foods’. Our findings identify a narrow conceptualisation of mealtimes focused on ‘health’ as limiting the potential to leverage mealtimes as places to support children’s nutrition and attendant development and learning.

Early education and care (ECEC) is part of the everyday life of the majority of children in developed economies^(1)^. Some children attend ECEC from their first year of life, while almost all children attend prior to school entry^(2)^. In Australia, 73 % of ECEC services serving children from birth to 5 years are centre-based childcare services and are attended for up to 12 h a day^(3)^. Children consume at least 50 % of their daily food intake^(4)^ when attending ECEC services, presenting exceptional opportunities to support nutrition and to teach and sustain lifetime patterns of food preference and eating behaviours^(5)^. For this reason, quality rating and improvement systems (QRIS) incorporate mealtimes and meal provision. Yet, the degree to which these QRIS systems capture current evidence pertaining to optimal provision, practices and nutrition education, and how these are captured in observations at assessment visits, is unknown. In this paper, we take the example of the Australian National Quality Framework (NQF) for ECEC and the associated national standard to ask whether these align with current literature on meal provision, practices and nutrition education. We then examine assessor field notes, collected as evidence during assessment and rating visits, to understand the ways in which food and mealtime practices are being assessed.

## Evidence for optimal provision, practices and nutrition education

Evidence from nutrition scholarship in the early years identifies three key knowledge domains with application in ECEC. First, the provision describes the quality and quantity of food provided by services. Second, mealtime practices encompass structure-related and autonomy-supportive feeding practices and educator-child interactions. Third, moments for learning entail opportunities for nutrition education during and outside of mealtimes.

Meal provision is important for physical and cognitive development in childhood^(6,7)^, influencing developing food preferences^(5)^, academic performance^(8)^ and averting the risk of chronic disease in adulthood^(9)^. Nutritionally balanced ECEC meals provide sustained energy^(10)^, enabling children to access educational opportunities throughout the ECEC day^(11)^. Nutritional deficiencies are a risk factor for suboptimal cognitive, social-emotional and motor development in children^(7)^. For example, the impact of iron deficiency anaemia before the age of two impacts school achievement^(12)^ and social-emotional development throughout childhood^(13)^. While deficiencies are less severe in high-income countries such as Australia, they are more prevalent among disadvantaged groups^(14)^. Prado (2014) identifies nutrition quality in early childhood as a mechanism to reduce socioeconomic disparities. Recent findings from Australia, however, identify that in practice, ECEC services may perpetuate nutrition inequity^(11,15,16)^.

ECEC mealtimes comprises strategies and behaviours utilised by services and educators during meals. Early mealtime experiences can shape food choices into adulthood^(17)^ with attendant effects on health and well-being^(18)^. Lifelong eating behaviours, appetite regulation and food preferences begin to form in childhood^(19)^, shaped by genetic factors^(20)^ and environmental influences such as meal structure^(21)^ and caregiver feeding practices^(22)^. For example, family style meals^(22)^, role-modelling eating^(23)^, repeat exposure to a variety of foods^(23)^ and the provision of non-coercive, autonomous environments, in which children choose how much to eat from what is offered, are strongly associated with more positive mealtime experiences, increased dietary quality and reduced fussy eating^(22,24)^. The benefits of mealtimes in ECEC, however, extend beyond nutrition, providing abundant opportunities for high-quality educator-child interactions. High-quality interactions, characterised by high levels of sensitivity, responsiveness and positive regard^(25)^, are fundamental for children’s social participation and educational achievement^(26,27)^. Interactions at mealtimes allow for rich educator-child conversations that strongly influence early language and cognitive development^(28–30)^ while also allowing for cross curricular learning and a sense of community^(31)^. The quality of educator-child interactions in ECEC are not consistent across the ECEC day. Higher quality interactions are seen during play than during routines such as meal and sleep times^(32)^.

Nutrition education is a widespread component of health promotion interventions in ECEC that aim to shape diet and eating behaviours^(33)^. Nutrition education *c*aptures strategies and environmental supports that facilitate diverse and nutritional food choices and behaviours conducive to health and well-being^(34)^. Evidence-based recommendations for specific components of nutrition education appropriate for the cognitive development of preschool children are lacking^(35)^. Moreover, evidence for interventions that increase children’s nutrition knowledge is low in quality and shows little positive effect on long-term dietary intake or food preferences^(36)^. Such limited effects are likely explained by the fact that young children are motivated by taste and curiosity, not healthfulness^(37)^. Increasingly, nutrition interventions that emphasise age-appropriate, experiential food opportunities, such as vegetable gardens and sensory food experiences, show promise, increasing children’s liking for and interest in nutritious foods^(35,38,39)^. The extent to which such different QRIS approaches capture nutrition education varies. For example, the Australian ECEC quality standard directs services to ‘actively promote healthy eating’ and ‘provide regular opportunities for explicit learning about health’^(40)^ but does not identify experiential nutrition. In contrast, Norway’s guidelines promote ‘pedagogical’ meals. Here, mealtimes are opportunities for broader education, including language (e.g. naming food and conversations), interactions, food skills (e.g. buttering bread), maths (e.g. weighing food), learning about the connection between food and health, sensory experiences and cultivating joy and curiosity in food^(41)^.

### Assessing early education and care provisions

**Figure 1.**
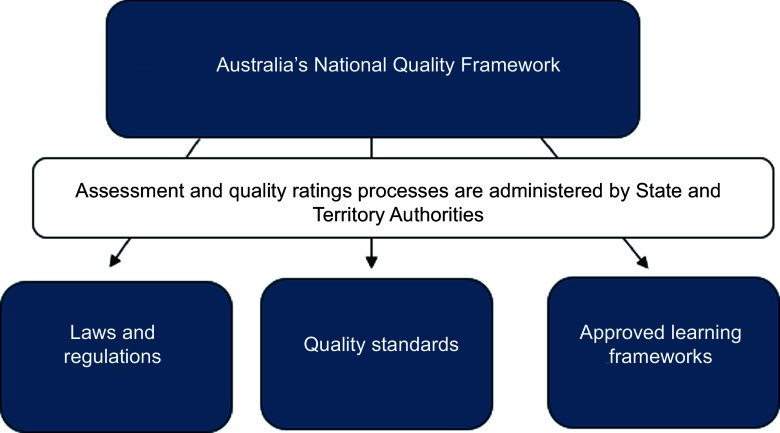
Figure to illustrate the components of the National Quality Framework in Australia.

Across developed nations, there is high investment in ECEC and an agenda for quality improvement^(1)^. To ensure that this investment is delivering on goals of supporting child development and health, QRIS are now commonplace across national jurisdictions^(42)^. These assessments of quality often include standards for food provision and mealtimes. In the present study, we examine the context of Australia. Australian Children’s Education and Care Quality Authority. Here, all licenced ECEC services must comply with the NQF^(40)^ (Fig. 1).

**Figure 2.**
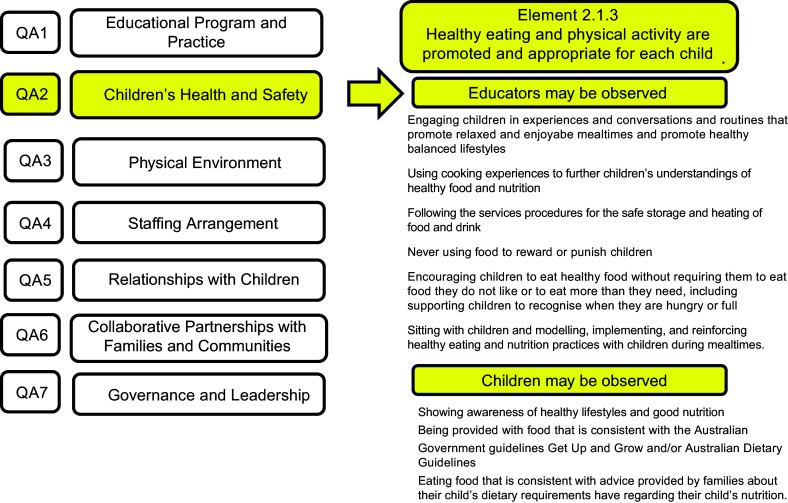
Figure to show Element 2.3.1 of Quality Area 2 within Australia’s National Quality Framework.

The Australian NQF is administered by state and territory authorised officers via an assessment and quality rating process. All ECEC services are assessed against the National Quality Standard (NQS) and National Laws and Regulations. The NQS comprises seven quality areas. Each quality area includes a set of standards and elements. Nutrition is featured in Quality Standard 2, Health and Safety (Fig. 2), and occupies one element (2.1.3), which states that ‘healthy eating and physical activity are promoted and appropriate for each child’ (NQF p152).

Under National Regulations, Australian ECEC services are required to practice safe food hygiene, provide access to drinking water and provide access to nutritious food based on individual dietary, religious or cultural requirements^(40)^. ECEC services are directed towards two main government publications for further nutrition guidance: *The Australian Dietary Guidelines*^(10)^ and the *Get Up & Grow* guidelines^(43)^. There is no requirement that services provide food for children; thus, some services require families to provide food.

In this study, through examination of detailed field notes made by authorised officers, those undertaking assessment of ECEC services, we ask:

1. what observational evidence on mealtimes in ECEC services is collected by those charged with undertaking assessment and rating of ECEC services; and
2. how does this evidence align with current knowledge pertaining to optimal nutrition in ECEC?

## Methods

### Data source

Randomised, de-identified field notes recorded by *n* 38 authorised officers during assessment and rating visits to ECEC services in Brisbane, Australia, in July 2018 were provided by the Queensland Government Department of Education. De-identified field notes from each authorised officer’s first visit, regardless of service type, in the month of October were provided to the research team. This strategy ensured data captured a random and diverse sample of ECEC services.

### Analysis

A total of 1748 field notes were available for ECEC services serving children aged birth to 5 years. Field notes for Out of Hours School Care (OHSC) and Family Day Care were excluded. Observations recorded for OHSC services within ECEC settings were retained. All field notes were checked for references to food or mealtimes and added to an Excel spreadsheet. These data were analysed using an abductive approach^(44)^ guided by key areas in the nutrition literature. The field notes were categorised into three categories mapping to the three theoretical domains of knowledge: provision, practices and nutrition education. Within these categories, data were read repeatedly and further coded, enabling the identification of data-driven themes with reference to existing evidence regarding mealtimes and nutrition in ECEC.

## Results

A total of 182 food/meal-related field notes were extracted from the data set and coded within the three theoretical domains of knowledge: *provisions, practices and education*. Table 1 outlines the themes within each domain. The number of themes within each domain exceeds the total number of field notes as themes were not exclusive and many field notes contained several themes.

**Table 1.** Themes developed from authorised officer field notes during assessment of early education and care services (excluding outside school hours services)

Domain	Theme	*n*	%	Description
Provision *n* 120 (42 %)	Food provision	45	38	Description of food provided to children (centre or family-provided). Includes discussions about menu development
	Hygiene and safety	40	33	Descriptions of food-safe practices such as wearing gloves.
	Water intake	15	13	Water is provided/accessible; children are reminded to drink water.
	Dietary requirements	13	11	Children’s dietary restrictions (allergies, religious beliefs, etc.) are considered when providing food or planning menus.
	Monitoring	7	6	Food intake is recorded.
Practices *n* 110 (39 %)	Routines	31	28	Descriptions of order of events and transitions.
	Independence	27	25	Independence at mealtimes is encouraged and/or facilitated, for example, self-service of food and self-feeding.
	Autonomy	26	24	Children share decisions about meal timings, how much to eat and what to eat.
	Educators sit/eat with children	12	13	Educators observed to sit and/or eat with children during a meal. Does include whether conversation or interactions also occurred.
	Environment	10	9	Creating a positive pleasant mealtime environment includes warm educator-child interactions. Tablecloths, relaxed conversations, etc.
	Feeding practices	3	3	Role modelling, pressure, restriction, encouragement to eat, etc.
Nutrition education *n* 51 (18 %)	Food talk	18	35	Neutral food talk at mealtimes. Includes general talk about food and the sensory properties of foods. Healthy talk is not included here.
	Food experiences	18	35	Food play, food exploration outside meals and stories including food themes.
	Sustainability	11	22	Environmentally responsible actions such as composting food scraps.
	Healthy talk	4	8	Food talk that focuses on the healthiness of food.

Note. Percentages are provided relative to the immediate superordinate category for each domain (% of all field notes) and theme (% of each domain).

### Domain 1: Provisions

‘Provision’ refers to types of foods provided to children, hygiene and food safety practices such as handwashing and food temperature and record-keeping. Many field notes described procedural functions:

‘Water – children have own individual water bottles avail on drink stand in room. Children observed taking bottles outside for morning tea also’. (Service 14)

‘Educators observed wearing gloves while feeding children bottles’. (Service 16)

Food provision was generally recorded as a list of items. Field notes sometimes described fruit or vegetables offered or available in lunchboxes but often lacked detail and did not describe meat/alternatives or quality of carbohydrates, resulting in an incomplete picture of food quality and absence of data on food quantity.

‘Kindy children observed being given sandwiches’. (Service 14)

Field notes relating to menus mostly confirmed that these were displayed in the service. One field note confirmed that ‘regular feedback from families, children and educators was sought’ in relation to the menu (service 17), and another noted that menus had not changed for a number of years:

‘Discussions with educators and the nominated supervisor confirmed that the menu was created a number of years ago through an app and had not changed in that time’. (Service 60)

Field notes also described whether services catered for dietary requirements such as food allergies:

‘A child in the toddler room was provided with bacon carbonara. Educators questioned each other as to if the child was able to have pork, to which one of the educators was not aware, and the child was served the bacon carbonara’. (Service 9)

Educators recording (i.e. record-keeping) food eaten by the children was also documented in field notes.

‘Children’s food intake is recorded and displayed for families’. (Service 4)

We note the absence of evidence in relation to food quantity, quality and children’s responses to food.

### Domain 2: Mealtime Practices

The domain ‘Practices’ describes educator mealtime behaviours or actions recorded by authorised officers and comprised the themes of routines, independence, autonomy, educators sitting with children, feeding practices and environment. Many field notes described routine activities at mealtime.

‘Children scrape their scraps into the bin and put their dirty dishes and cutlery in a tub for washing’. (Service 45)

Many field notes also referred to activities or situations that facilitate or hinder children’s independence in serving or eating food.

‘Educator supported children to use a piece of equipment to peel their apples’. (Service 1)

‘The tongs were adult sized and difficult for the children to manipulate’. (Service 33)

Other notes identified the promotion of autonomy, describing observations of children’s active participation regarding mealtimes and food.

‘Children share decisions about meal timings, how much to eat, what to eat’. (Service 1)

Educators sitting with children was a less frequently recorded practice. Some authorised officers noted educators sitting or eating with children but did not provide specific information about the interactions between educators and children that occurred.

‘Educators sitting with children at food times, supervising’. (Service 18)

A small number of field notes provided additional detail about conversations between educators and children or when there was a lack of conversation or interaction observed.

‘Educators sat with the children whilst they ate, however, limited conversation was instigated or promoted by the educators. For example, two educators spoke in their native language during this time’. (Service 28)

In a few notes, the tone of the mealtime environment was described. This theme also included field notes that described warm educator-child interactions.

‘The service follows a progressive meal practice to support children with their independence and to give children the opportunity to connect with each other and their educators in a relaxed atmosphere’. (Service 49)

One field note described a less positive mealtime environment.

‘In some rooms, children were observed sitting at tables for long periods of time for meals to be served. Limited conversations between educators and children occurred during this time’. (Service 9)

The smallest theme within ‘Practices’ noted when educators utilised the feeding practices of role-modelling and encouragement to eat during mealtimes.

‘Educators provide role modelling for children, eating the food also on offer’. (Service 4)

Field notes regarding observations of children’s experiences and behaviours at mealtimes are absent.

### Domain 3: Nutrition Education

Education was notably a domain in which there were the least amount of field notes identified. In the limited number of examples, some form of nutrition education (verbal or experiential) both within and outside of mealtimes was documented. Field notes in this theme described food talk, conversations about the properties of food and sometimes linking food to children’s prior knowledge and experiences.

‘Discussions around where does milk come from. The conversation went onto orange juice and how this is made. Educator prompted the children by asking them how they previously made orange juice and what fruit they used to make it’. (Service 12)

Food experiences outside of mealtimes were noted by some authorised officers. These included children interacting with edible gardens, play food (Play-Doh, etc.), art, sensory food experiences, stories and role play.

‘Educator responds to children’s interest in making food from playdough by getting play food out for the children, children responding with excitement educator asking children what each food item is’. (Service 18)

‘Educator sitting with the children at the spaghetti station discussed how the spaghetti felt on their hands – was it smooth, hard, cold or hot? Educator encouraged the children to squeeze the spaghetti between their fingers’. (Service 12)

The Education theme also included sustainable practices. All but one field note in this theme referred to the practice of putting food scraps into compost bins. One field note observed plastic free lunchboxes.

The final Education theme comprised field notes about overt educator attempts to teach children to recognise and eat ‘healthy’ food.

‘What healthy food do we eat? Broccoli, apples, pancakes and jelly is that healthy? No it’s a sometimes food’. (Service 6)

‘What healthy choices do you have today? Children ate yogurt, sandwiches. Healthy (Educator) said holding up sandwich. Yep, that is healthy’. (Service 15)

## Discussion

Nutrition is a prerequisite for learning and thriving. In early childhood, access to adequate nutrition, responsive feeding practices and positive learning experiences with and about food are important experiences that set the foundations of lifetime well-being and productivity. Each, therefore, should be part of the everyday experiences in ECEC and a component of the assessment of ECEC quality. Accordingly, our study sought to examine whether current policy and practice in defining and assessing nutrition quality in ECEC capture domains of quality identified in the nutrition literature. Our study utilised unique data, a random sample of field notes from authorised officers (*n* 38) undertaking assessment ratings of ECEC services. Our analysis of these data asked two important questions:

1. What observational evidence on mealtimes in ECEC services is collected by those charged with undertaking the assessment and rating of ECEC services; and
2. how does this evidence align with current knowledge pertaining to optimal mealtimes in ECEC?

The body of child nutrition evidence identifies three knowledge domains that define quality in the context of ECEC: provision, practices and education^(33)^. Analysis of the field notes made by authorised officers undertaking quality assessment in ECEC identified that the evidence collected closely aligned to the content of the National Quality Framework. As a consequence, their records focused on compliance with health and safety requirements. The findings identify a problem of omission. Notes on nutritional quality and quantity of food, feeding practices and nutrition education were limited.

Despite the importance of food quantity and quality^(10)^, notes relating to *‘Provision’* rarely provided such records. Rather, field notes documented hygiene practices such as hand washing and children’s access to water and whether menus were appropriate and displayed in services. These observations align with the current standards (2.1.3). Thus, the authorised officers were fulfilling their requirements in observation, however, the need to modify the standard to include observation of food quantity and quality is indicated. Assessment of dietary quality in ECEC varies between countries. In Australia, while there is not a requirement to directly assess the quality or quantity of food provided, there is evidence that food provision in Australia’s ECEC services does not meet national dietary recommendations^(15,45)^ and can affect learning opportunities^(11)^. The current requirement to provide written menus does not provide sufficient evidence that food provided by a service is both nutritious and adequate in quantity. In contrast, in the US state of California, services that receive Child and Adult Care Food Program funding are directly monitored via site visits three times per year to ensure meals meet optimal nutritional standards^(46,47)^.

Interactions between educators and children at mealtimes play a significant role in nutrition education. Yet, the field notes captured very little evidence about interactional quality and nutrition education, and the degree of detail varied across different assessors. For example, ‘*educators sitting with children at food times supervising’ (service 18)* lacks sufficient detail to assess the educational opportunities taken at mealtimes. In contrast, some assessors noted that educational opportunities were taken or missed. Notable are examples of records describing children’s opportunity for autonomy and independence at mealtimes, expanding beyond a narrow health focus to include developmental opportunities. Mealtime interactions provide an environment ideal for developing brain architecture and future well-being^(48)^. Yet, a dominant health lens is present in our analyses of field notes. In ECEC, positive emotional interactions are a strong indicator of quality but are often observed as of lower quality at routine times, particularly mealtimes^(11,32)^. Our data suggest mealtimes may well be ‘barometer events’ that distinguish services that take educational opportunities and those that do not. The implications for those undertaking assessment are that these are times to observe beyond hygiene and health to include interactions – mealtime moments that matter.

Our analysis identified that among the assessor’s field notes relating to food, less than a fifth described any form of nutrition education. Our data are assessor field notes and may not reflect educators’ behaviours in this domain. They do however reflect the low priority placed on opportunities for mealtimes, and nutrition more broadly, to be a focus for learning in guides to assessment and attendant assessment process.

### Strengths and limitations

QRIS in ECEC drive practices. The content of the standards they assess are therefore potent in directing the behaviour of ECEC provider organisations and educators within services. In this study, we focused on mealtimes as a source of information in the assessment and rating of ECEC service quality. The strength of our study is in providing a unique insight into policy conceptualisations of quality and their limitations. Here we show, in the case of Australia, a limited conceptualisation that focuses on ‘healthy food’ without consideration of the social context of the children who attend services, the mode of food provision (centre or family-based) and educator’s knowledge base. A key message is that mealtimes and food present exceptional opportunities to assess ECEC quality but are not necessarily conceptualised as vital places for learning and development.

The study must be viewed in terms of intent and limitation. Our aim was to examine how QRIS processes work to define and assess ECEC food provision, practices and nutrition education. While QRIS processes aim to capture a representation of the quality of an ECEC service, the field notes we analysed are limited to the time of data collection and may not fully capture all activities relating to food in the services assessed. Australia’s NQS has been updated since the data for this study were collected. However, changes to the element relating to nutrition were minimal and did not extend the assessment of mealtimes. The field notes reflect the assessor’s response to the quality standard, their training and subsequent interpretation of what should be assessed, while the behaviours of the service during a notified inspection may also be tailored to their expectations set by the quality standard.

### Implications for research and practice

This analysis of field notes taken in the Assessment and Ratings of ECEC services in Australia provides a window into how quality is operationalised in observations of mealtimes. Our data suggest that the policy, captured in quality standards, drives the content of data collections when assessing and rating an ECEC service. Such ratings are important in a system that is a competitive market in which ratings can drive parent choice. Our data identify a narrow conceptualisation of mealtimes focused on ‘health’ as limiting the potential to leverage mealtimes as places to support children’s development and learning. Development and capacity to learn are affected by the quantity and quality of food provided^(7,49)^, yet these are not explicitly documented as a focus of the current definition of ECEC quality in the Australian example provided here. This is a concern. Recent Australian data identify that the most disadvantaged children are those least likely to have adequate quantity and quality of food when attending ECEC^(15)^. The case for the provision of sufficient levels of nutrition is strong, but not within the quality standard. Beyond, we argue that mealtimes are ‘barometer events’ in which opportunities for learning are not often taken but, when they do, are a marker of quality. Considering mealtimes as integral to the ECEC curriculum and not just a functional routine is an important component of delivering ECEC quality. As QRIS processes drive service and assessor behaviours, those nations that do not currently focus beyond ‘healthy choices’ might look to the examples in which food provision and mealtimes are seen as educational opportunities not only for nutrition education but are developmental opportunities.
